# The Spemann organizer meets the anterior-most neuroectoderm at the equator of early gastrulae in amphibian species

**DOI:** 10.1111/dgd.12200

**Published:** 2015-03-10

**Authors:** Takanori Yanagi, Kenta Ito, Akiha Nishihara, Reika Minamino, Shoko Mori, Masayuki Sumida, Chikara Hashimoto

**Affiliations:** 1 JT Biohistory Research Hall1-1 Murasaki-cho, Takatsuki, Osaka, 569-1125, Japan; 2 Department of Biological Sciences, Graduate School of Science, Osaka UniversityToyonaka, Osaka, 560-0043, Japan; 3 Institute for Amphibian Biology, Hiroshima UniversityKagamiyama, Higashi-Hiroshima, Hiroshima, 739-8526, Japan

**Keywords:** amphibian, chordate, gastrulation, movement, Spemann organizer

## Abstract

The dorsal blastopore lip (known as the Spemann organizer) is important for making the body plan in amphibian gastrulation. The organizer is believed to involute inward and migrate animally to make physical contact with the prospective head neuroectoderm at the blastocoel roof of mid- to late-gastrula. However, we found that this physical contact was already established at the equatorial region of very early gastrula in a wide variety of amphibian species. Here we propose a unified model of amphibian gastrulation movement. In the model, the organizer is present at the blastocoel roof of blastulae, moves vegetally to locate at the region that lies from the blastocoel floor to the dorsal lip at the onset of gastrulation. The organizer located at the blastocoel floor contributes to the anterior axial mesoderm including the prechordal plate, and the organizer at the dorsal lip ends up as the posterior axial mesoderm. During the early step of gastrulation, the anterior organizer moves to establish the physical contact with the prospective neuroectoderm through the “subduction and zippering” movements. Subduction makes a trench between the anterior organizer and the prospective neuroectoderm, and the tissues face each other via the trench. Zippering movement, with forming Brachet's cleft, gradually closes the gap to establish the contact between them. The contact is completed at the equator of early gastrulae and it continues throughout the gastrulation. After the contact is established, the dorsal axis is formed posteriorly, but not anteriorly. The model also implies the possibility of constructing a common model of gastrulation among chordate species.

## Introduction

Spemann and Mangold found that the dorsal blastopore lip of an amphibian gastrula can induce a secondary axis when transplanted into another embryo's ventral side (Spemann & Mangold 1924). The dorsal blastopore lip was named as “the organizer” because it induces the differentiation of cells that attach to it into well-organized axial structures (such as head, tail, and neural tissue).

Gastrulation is a critical stage in the formation of the central nervous system. Neurulation of the dorsal ectoderm is regulated by the axial mesoderm, known as the organizer which includes prechordal plate and the notochord. In the amphibian gastrulation process, the axial mesoderm, which is derived from the dorsal marginal zone, invaginates, involutes into the body, and then migrates toward the animal pole on the inner surface of the blastocoel roof. The inner surface of the blastocoel roof is ready for the directional migration of the leading edge along the surface of fibronectin substrate (Nakatsuji *et al*. 1982; Boucaut *et al*. 1984; Winklbauer & Nagel 1991; Johnson *et al*. 1993), and the migration was observed *in vivo* and *in vitro* (Hara *et al*. 2013; Moosmann *et al*. 2013).

**Figure 10:**
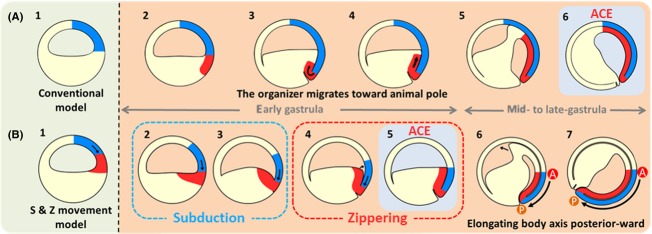
Conventional model and Proposed model of amphibian gastrulation. (A) Conventional mode of amphibian gastrulation. (B) Proposed model of amphibian gastrulation “S&Z movement”. The organizer and the prospective neuroectoderm are represented by red and blue, respectively. (A-1) Blastula embryo. (A-2, 3, 4, 5) The organizer which is derived from the dorsal marginal zone, invaginates, involutes into the body, and then migrates toward the animal pole on the inner surface of the blastocoel roof. (A-6) Anterior contact establishment (ACE) occurs around the animal pole area of mid- to late-gastrula embryo. (B-1) Blastula embryo. (B-2,3) The embryos undergo subduction movement. (B-4, 5) The zippering movement leads to physical contact between the blastocoel floor and the blastocoel roof. ACE occurs at equatorial region of very early gastrula. (B-6) The leading edge tissue migrates animally beyond the region of anterior contact. (B-7) An axial structure is progressively formed toward the posterior during the rest of the gastrulation movement. Curved arrows between A and P indicate the direction of axial structure formation. 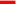, organizer; 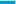, prospective neuroectoderm.

Anterior terminal neural tissue is determined when the anterior tip of the axial mesoderm reaches the anterior-most portion of the prospective neuroectoderm at the blastocoel roof. In this work, the establishment of this physical contact is called “anterior contact establishment” (ACE) (*cf*. Fig. 10A-6). The establishment of physical contact between the organizer and the prospective neuroectoderm is one of the important steps for axis formation. As the prospective head neuroectoderm is known to locate around the animal pole in fate maps of early gastrula (Vogt 1929; Nakamura 1942; Keller 1975), ACE should occur around the animal pole area of mid- to late-gastrula embryo.

However, we found that gastrulation movement in *X. laevis* is distinct from this conventional model of amphibian gastrulation (*cf*. Fig. 10A) (Koide *et al*. 2002). In that report, we suggested that ACE occurs at the equator of early gastrula, and the physical contact of the anterior organizer with the prospective head neuroectoderm continues throughout the gastrulation process. The leading edge tissue migrates animally beyond the region of anterior contact after ACE. Our model was supported by live imaging of a single embryo using microscopic magnetic resonance imaging (MRI; Papan *et al*. 2007). It is still unknown whether the *X. laevis* gastrulation movement is applicable to the movement of other amphibians because it is believed that the features of gastrulation vary among species (Shook *et al*. 2002; Moya *et al*. 2007; del Pino *et al*. 2007).

**Figure 9:**
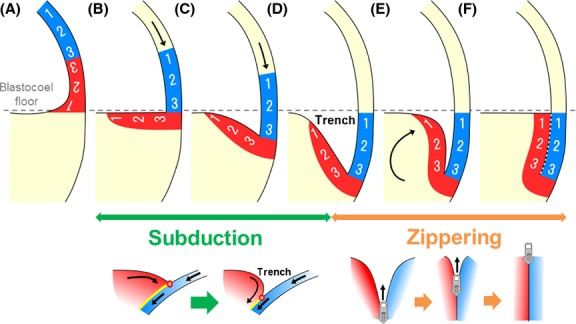
Model of “subduction and zippering” movement. The organizer and the prospective neuroectoderm are represented by red and blue, respectively, and the future A-P axis is indicated by numbers. The grey broken line indicates the blastocoel floor (equatorial) level of the embryo. (A) The organizer and the prospective neuroectoderm are aligned in tandem in the dorsal blastocoel roof at the blastula stage. (B–D) The “subduction” movement. (B) The prospective neuroectoderm (blastocoel roof) and the anterior organizer (blastocoel floor) are at right angles to each other at the onset of gastrulation. (C) The blastocoel floor is pushed down by the epiboly-like movement of the blastocoel roof (arrows). (D) The anterior organizer is dragged down, and the movements lead to the formation of a trench with the prospective neuroectoderm. (D–F) The “zippering” movement. (D) The anterior organizer and the prospective neuroectoderm face each other with the trench in between. (E) The red side of the trench approaches the blue side by vegetal rotation-like movement (curved arrow). (F) Then, the anterior organizer and the prospective neuroectoderm make physical contact, and Brachet's cleft (white broken line) is formed. The conceptual diagram of the “subduction and zippering” movement is shown below. Note that if the arrangement of the cells does not change in the region, the organizer seems to turn around during gastrulation (compare panel [A] with panel [F]). The dorsal lip is known to contribute to the pharyngeal endoderm (Vogt 1929; Nakamura 1942; Keller 1975; and see below). 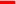, organizer; 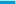, prospective neuroectoderm.

Here, we show that the *X. laevis* model is fundamentally applicable to a wide variety of amphibian species, and propose a unified model of amphibian gastrulation movement (*cf*. Fig. 10B). In the model, the anterior organizer is present at the blastocoel floor at the onset of gastrulation, and moves to the equator to make physical contact with the prospective head neuroectoderm through the “subduction and zippering” (S&Z) movement (*cf*. Fig. 9) during the early step of gastrulation. The blastocoel roof becomes not neural but epidermal tissue after the contact. This physical contact continues to the end of gastrulation movement, indicating that the head is fixed at the dorsal equator of early gastrula so that the A-P axis is formed toward the posterior as the posterior organizer involutes inward (*cf*. Fig. 10B-6,7). This model of amphibian gastrulation would enable us to make a direct comparison with the gastrulation movements of other chordate species.

## Materials and methods

### Adults

*Xenopus laevis* purchased from Watanabe Zoushoku (Hyogo, Japan) and *Cynops pyrrhogaster* collected in Hyogo, Japan were maintained in JT Biohistory Research Hall (Osaka, Japan). *Rana japonica* was collected in Kagoshima and Hiroshima, Japan. *Odorrana supranarina* was collected in Okinawa, Japan. *Silurana tropicalis* was maintained by the Institute for Amphibian Biology (Hiroshima University) through the National Bio-Resource Project of MEXT, Japan. The other amphibians were kept by the strain maintenance team of the Institute for Amphibian Biology.

### Embryos

Embryos of *R. japonica, Rana porosa brevipoda, X. laevis,* and *S. tropicalis* were obtained by *in vitro* fertilization. Embryos of the other species were obtained by natural mating. The jelly coat was removed chemically with 1.5% thioglycolic acid (pH 9.5) and/or removed mechanically. The embryos were kept in 0.1× Steinberg's solution (58 mmol/l NaCl, 0.67 mmol/l KCl, 0.44 mmol/l Ca[NO_3_]_2_, 1.3 mmol/l MgSO_4_, 4.6 mmol/l Tris, pH 7.8) until they reached the desired stage at 13°C for *X. laevis*; at 19°C for *R. porosa brevipoda*; at 20°C for *Ambystoma mexicanum, Bombina orientalis,* and *Cynops ensicauda*; at 22°C for *S. tropicalis*; at 23°C for *C. pyrrhogaster*; and at 18°C for the other amphibians. Staging of gastrulae was performed according to the time length after the time of blastopore appearance. Note that we raised *X. laevis* embryos at low temperature (13°C) to determine the embryonic stages exactly. In this condition, gastrulation completes in 20 h. The time of blastopore appearance was defined by the appearance of a pigment line formed by the accumulation of bottle cells. As the need arises, the embryos were fixed with 10% formalin in PBS (175 mmol/l NaCl, 7.4 mmol/l Na_2_HPO_4_, 1.9 mmol/l NaH_2_PO_4_, pH 7.8) at the indicated stages, and were sagittally dissected with a microtome blade.

### Staining of inner surface of blastocoel cavity

For internal labeling, 20–250 nl (depending on egg size) of 1% neutral red solution in saline was injected into the blastocoel cavity at the indicated stages in 3% Ficoll/0.5× Steinberg's solution. Then, the embryos were transferred to 0.5× Steinberg's solution without Ficoll where they developed into neurulae.

### Labeling of external surface

Vital dye staining of the equatorial region of the embryos was performed as described previously (Kaneda *et al*. 2009) with minor modifications. To precisely stain tissue lined by the leading edge tissue, we measured the distance between the leading edge tissue and the dorsal lip of blastopore at 7 h after blastopore appearance (ACE) in the sagittal sections of *C. pyrrhogaster* embryos. The distance was 1.3 mm on average. Then, superficial vital dye staining of *C. pyrrhogaster* embryos was performed at the distance of 1.3 mm from the dorsal lip using an ocular micrometer in 0.1× Steinberg's solution.

### Blastocoel roof-less embryos

The blastocoel roof was removed at the level of the blastocoel floor with fine forceps and eyebrow knives at the indicated stages. The blastocoel roof-less embryos were developed in 0.5× Steinberg's solution containing 100 μg/ml kanamycin.

### Graft assay

The dorsal blastopore lip at blastopore appearance or the blastocoel floor at ACE was detached from the embryos with eyebrow knives, and grafted to the prospective ventral side of the blastopore-appearance-stage recipient embryos by the Einsteck method (Slack & Isaacs 1994). The recipient *X. laevis* embryos were allowed to develop in 1× Barth's solution containing 100 μg/ml kanamycin at 18°C. The recipient *C. pyrrhogaster* embryos were allowed to develop in 1× Steinberg's solution containing 25 units/ml penicillin G and 25 μg/ml streptomycin and 100 μg/ml kanamycin at 20°C.

### Preparation of probes

*Xenopus laevis* and *C. pyrrhogaster chordin* probes (Yokota *et al*. 1998) were synthesized from cDNA independently cloned from each gastrula cDNA library in pCS2AT+, linearized with EcoRI, and transcribed from the T7 promoter.

### *In situ* hybridization

Whole-mount *in situ* hybridization was performed as reported (Hemmati-Brivanlou *et al*. 1990). Section *in situ* hybridization was performed essentially as described (Butler *et al*. 2001) with the following modifications. The embryos were fixed for 2 h in MEMFA for *X. laevis* and 4% paraformaldehyde in PBS for *C. pyrrhogaster*. The embryos were sectioned (10 μm thickness) and expanded in the water bath (Section Transfer System; Thermo, MA, USA). The sections were arranged in layers on MAS-coated glass slides, and left to dry overnight on a hot plate set at 40°C. As the prehybridization treatment, the slides were rinsed with 1× PBST. The RNase treatment subsequent to the hybridization was omitted, and probe washes were carried out at 60°C with 5× standard saline citrate (SSC) and 2× SSC three times for 10 min each, respectively, and then at room temperature with 1× TBST three times for 5 min each.

### Time-lapse microscopy

For time-lapse microscopy, the embryos were embedded in 10% gelatin/0.1× Steinberg's solution, cut out as cuboids with gelatin, and transferred to a new dish containing 0.1× Steinberg's solution with their positions fixed. Photomicrographs were taken every 10 min using a digital optical microscope (VHX-200; Keyence, Osaka, Japan) in a temperature-controlled room at 18–22°C.

## Results

### Prospective head neuroectoderm makes physical contact with leading edge tissue at early gastrula stage

**Figure 1:**
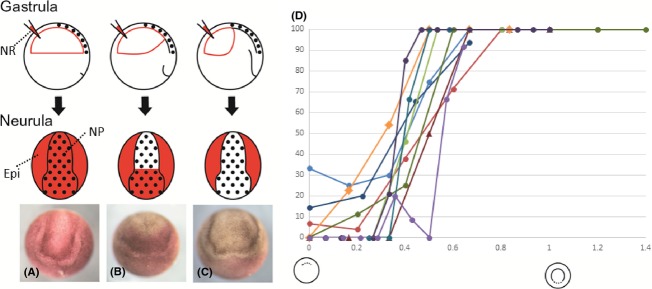
Comparison of timing when leading edge tissue covers inner surface of entire prospective neuroectoderm among amphibian species. (A–C) Schematic representation of neutral red labeling experiment. Blastocoels in embryos at the gastrula stage were injected with neutral red. Once the blastocoel roof contacted the inner tissue directly, the area of contact would not be stained by neutral red. When they grow up to neurulae, the stained state of the neural plate would enable us to know how the inner tissue lined the neuroectoderm at the injection time. The stippled area represents the prospective neuroectoderm (top panel) or the neural plate (middle panel). Bottom panels show *Hynobius nebulosus* neurulae whose blastocoels at the gastrula stage were injected with neutral red. The neural plate was entirely stained (type-A, A); the anterior portion of the neural plate was stained (type-B, B); no staining of the neural plate took place (type-C, C). Note that type-C includes embryos whose neural crests were labeled without neural plate staining. Epi, epidermis; NR, neutral red, NP, neural plate. (D) Graph of percentages of type-C neurula embryos in various amphibian species. In the horizontal axis, the injection time is expressed as the ratio of the length of time of blastopore appearance (“0”) to that of circular blastopore pigment line formation (“1”). Graph legends are shown on the right side. The number of embryos examined each time is shown in Table1. 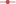, *Bombina orientalis*; 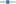, *Rana japonica*; 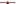, *Rana porosa brevipoda*; 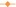, *Rana rugosa*; 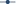, *Silurana tropicalis*; 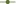, *Xenopus laevis*; 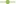, *Ambystoma mexicanum*; 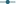, *Cynops ensicauda*; 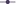, *Cynops pyrrhogaster*; 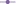, *Hynobius nebulosus*.

As the first step toward understanding the mechanism of amphibian gastrulation, we examined when the physical contact between the organizer and the prospective head neuroectoderm is established in various amphibian species. We analyzed anuran amphibian embryos of six species belonging to three families: fire-bellied toad *B. orientalis*, Japanese brown frog *R. japonica*, Nagoya daruma pond frog *R. porosa brevipoda*, wrinkled frog *Rana rugosa*, *X. laevis,* and Western clawed frog *Silurana tropicalis*. We also observed the gastrulation of urodelan amphibian embryos of four species belonging to three families: Mexican salamander *A. mexicanum*, sword-tail newt *C. ensicauda*, Japanese fire belly newt *C. pyrrhogaster,* and Japanese clouded salamander *Hynobius nebulosus*. We labeled the inner surface of blastocoel cavities of the embryos at various gastrula stages with neutral red (Fig.1A–C). This labeling allows us to determine the extent of the physical contact between the leading edge tissue and the prospective head neuroectoderm, by observing the embryos at the neurula stage. We categorized the labeled neurula embryos into three types. In type-A embryo, the neural plate is entirely stained with vital dye, which means that the entire prospective neuroectoderm is still in the blastocoel roof when labeled (Fig.1A). In type-B embryo, the anterior portion of the neural plate is stained, which means that the leading edge tissue is on the way to the anterior end of the prospective neural region when labeled (Fig.1B). In type-C embryo, no neural plate staining is observed, which means that the inner tissue lines the entire prospective neuroectoderm when labeled (Fig.1C). Therefore, type-C embryo is characterized by the establishment of physical contact at the neurula stage (Table1).

**Table 1:** Number of embryos receiving neutral red injection into blastocoel roof at each time

Injection time	*Bombina* orien talis	Rana japonica	*Rana porosa* brecipoda	*Rana rugosa*	*Xenopus laevis*	*Xenopus* tropicalis	*Ambystoma mexicanum*	*Cynops* ensicauda	*Cynops pyrrhogaster*	*Hynobius* nebulosus
0	15	3	5	14	54	21	7		7	16
0.33						20				
0.66						26				
1	25	4	5	22	27	16			2	7
2	29	10	8	24	24		7		14	15
3	35	8	10	16	52					13
4	23	5	8	20	33		12		20	10
5	13		8	20	40				19	10
6	14	5	8		20		13	2	27	12
7					19				6	11
8							13	4	12	12
9										12
10							14	3	4	
12							7	2		
13									2	
14								3	2	
15									2	
(hour after BA)										
Time of circular blastopore pigment line (hour after BA)	5	6	6	6	5	1.5	15	24	15	14

To know when the leading edge tissue reaches the anterior-most portion of neural tissue, we examined when the vital dye should be injected to make the type-C embryo. The results are summarized in Figure1D (detailed results are shown in Fig. S1). To accommodate the difference in developmental speed among species, the horizontal axis in Figure1D indicates the ratio of the length of time of blastopore appearance (“0”) to that of circular blastopore pigment line formation (“1”). At the time length ratios of 0.4–0.6, the rate of type-C embryo emergence exceeded 70% in all the species examined. The formation of the circular blastopore is considered to be the onset of the mid-gastrula stage (Keibfl 1925; Okada & Ichikawa 1947; Nieuwkoop & Faber 1956). In fact, it takes a very short time from the blastopore appearance to ACE (ACE occurs in the first 3 h in the whole 20-h process of gastrulation in *X. laevis*, and 7 h in 24-h in *C. pyrrhogaster*). Taken together, those results indicate that the establishment of physical contact between the leading edge tissue and the anterior neuroectoderm occurs at the early gastrula stage. In other words, blastocoel roof at this stage does not contribute to future neural tissue.

### Equatorial region at ACE ends up to anterior-most neural plate

**Figure 2:**
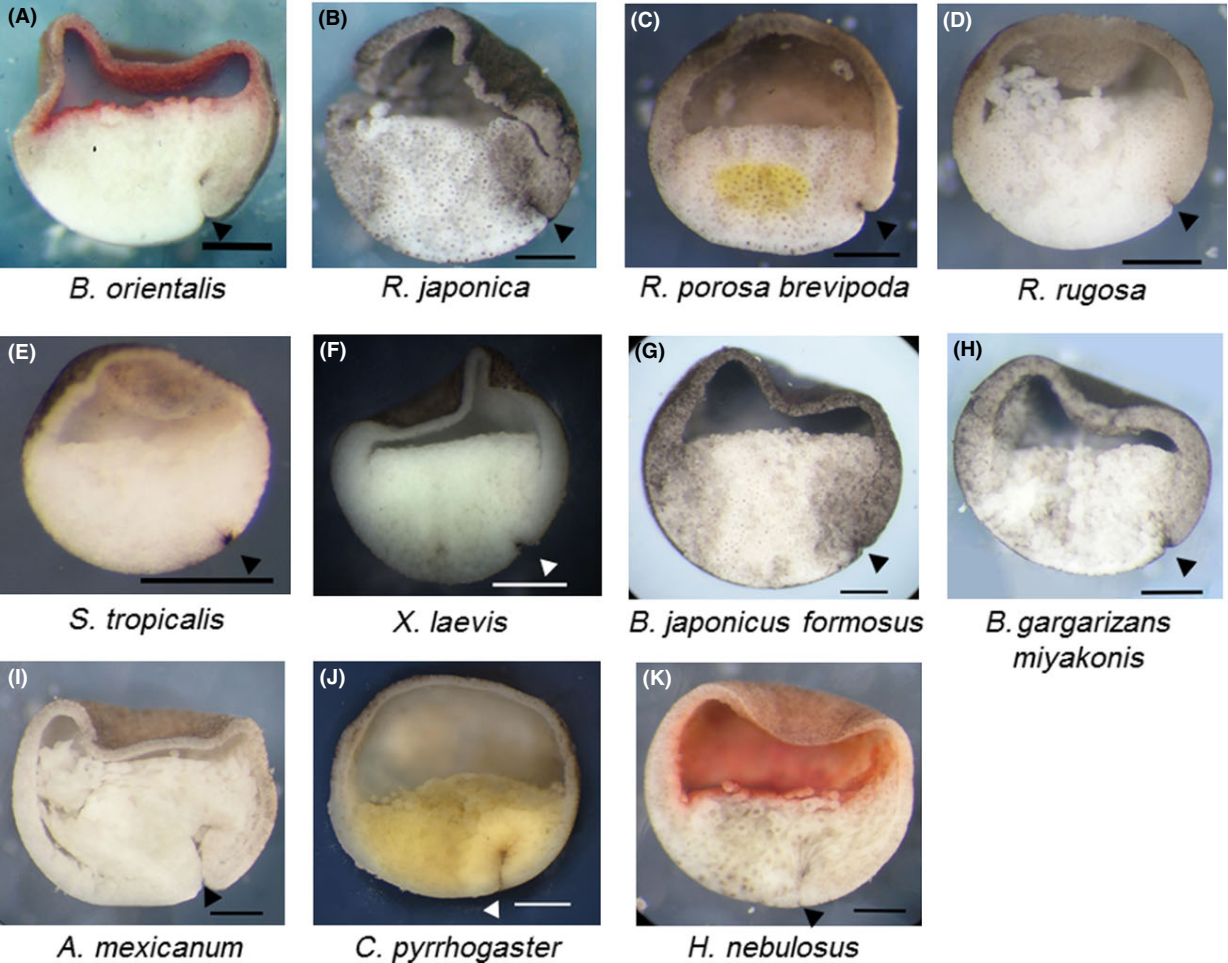
Leading edge tissue reaches anterior-most prospective neuroectoderm at equatorial region. Internal morphology of embryo at ACE. Blastopore is indicated by a black arrowhead. (A) *Bombina orientalis* at 4 h after blastopore appearance, (B) *Rana japonica* at 4 h after blastopore appearance, (C) *Rana porosa brevipoda* at 4 h after blastopore appearance, (D) *Rana rugosa* at 3 h after blastopore appearance, (E) *Silurana tropicalis* at 60 min after blastopore appearance, (F) *Xenopus laevis* at 3 h after blastopore appearance, (G) *Bufo japonicus formosus* at 4 h after blastopore appearance, (H) *Bufo gargarizans miyakonis* at 4 h after blastopore appearance, (I) *Ambystoma mexicanum* at 8 h after blastopore appearance, (J) *Cynops  pyrrhogaster* at 7 h after blastopore appearance, and (K) *Hynobius nebulosus* at 9 h after blastopore appearance in gastrula embryos. (A–F, I–K) The embryo was cut sagittally. The leading edge tissue is located in the equatorial region and the embryo has Brachet's cleft. (G, H) The embryo was fixed when Brachet's cleft was formed. The leading edge is located at the equatorial region. It may be important to mention that embryos of Urodele species have relatively long archenteron at anterior contact establishment (ACE), though anurans only have a dent at blastopore. This may be caused by a difference of gastrulation movement between them.

**Figure 3:**
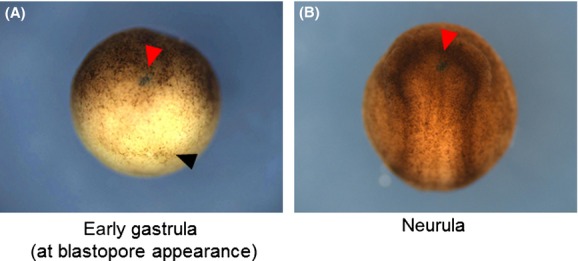
Vital dye staining of equatorial outer surface in *Cynops pyrrhogaster*. *C. pyrrhogaster* neurula with Nile blue staining of the equatorial surface at 7 h after blastopore appearance. The labeled region is indicated by a red arrowhead. (A) Immediately after labeling. (B) Labeled cells are localized in the anterior-most neural plate (15/25). Black arrowhead in panel A indicates blastopore.

To observe the internal morphology of the embryos after physical contact was established, we fixed the embryos at ACE and cut them along the mid-sagittal plane (Fig.2A–F,I–K). Figure2 shows that the leading edge is located at the equatorial region in all the embryos. This suggests that the prospective head neuroectoderm is located at the equatorial region at ACE. If this were so, the ectoderm associated with the leading edge around the dorsal equatorial region at ACE would end up as the anterior-most neural tissue, such as the forebrain. To confirm this possibility, we labeled the dorsal equatorial region (tissue precisely lined by the leading edge) of *C. pyrrhogaster* at ACE, and examined whether the labeled tissue contributes to the anterior portion of the neural plate. As a result, the labeled cells (Fig.3A) were found in the anterior-most neural plate (Fig.3B). In combination with our previous result (Koide *et al*. 2002), it seems that the ectodermal layer on the equator at ACE corresponds to the prospective head neuroectderm both in anuran and urodele species.

### The blastocoel roof at ACE does not contribute to neural tissue but contribute to epidermis

**Figure 4:**
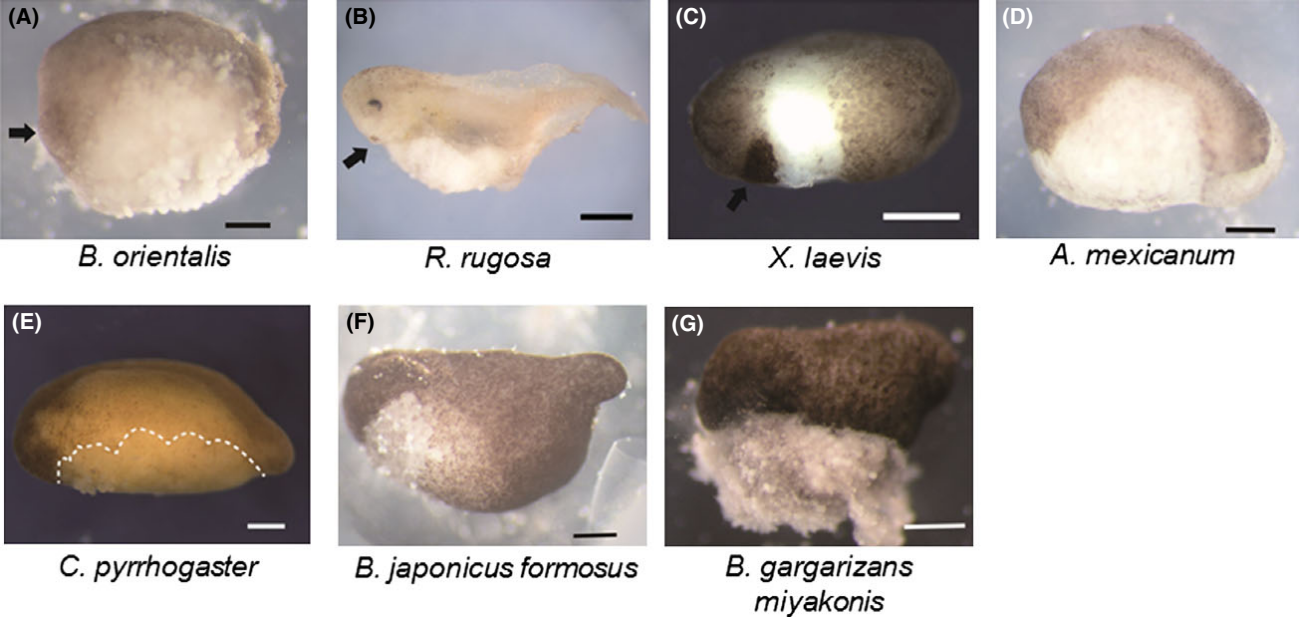
Blastocoel roof after anterior contact establishment (ACE)is dispensable for neural formation. Tailbud embryos whose blastocoel roof was removed at the gastrula stage. Lateral view. Head is left. (A) *Bombina orientalis*, (B) *Rana rugosa*, (C) *Xenopus laevis*, (D) *Ambystoma mexicanum*, (E) *Cynops pyrrhogaster*, (F) *Bufo japonicus formosus*, and (G) *Bufo gargarizans miyakonis*. (A–E) The blastocoel roof was removed at ACE. Black arrows in A–C indicate cement gland. Broken line in (E) indicates the border of epidermis. (F, G) The blastocoel roof was removed 4 h after blastopore appearance when Brachet's cleft was just formed.

**Figure 5:**
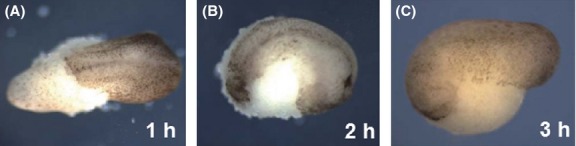
Formation of neural tissue is influenced by blastocoel roof removal time in *Xenopus laevis*. The blastocoel roof was removed 1, 2, and 3 h after blastopore appearance. The resultant embryos showed some differences during development to the tailbud stage. (A) The blastocoel roof was removed 1 h after blastopore appearance. The embryo has only a posterior dorsal axis. (B) The blastocoel roof was removed 2 h after blastopore appearance. The embryo has an anterior axis but no head. (C) The blastocoel roof was removed 3 h after blastopore appearance (ACE). The embryo has whole dorsal axis including cement gland.

From these observations, we hypothesized that the blastocoel roof at ACE is dispensable for the formation of dorsal structures. To check this hypothesis, we removed blastocoel roofs at ACE in *R. rugosa, X. laevis*, *B. orientalis*, *C. pyrrhogaster,* and *A. mexicanum* embryos*,* and monitored how the embryos would develop. In all the blastocoel roof-less embryos, dorsal structures, including complete head structures, showed normal development, but the ventral epidermises were absent (Fig.4A–E 3/3 in *B. orientalis B. orientalis*, 4/5 in *R. rugosa,* 17/20 in *X. laevis*, 5/6 in *A. mexicanum*, and 5/5 in *C. pyrrhogaster*). In addition, we recognized the cement gland in the blastocoel roof-less anuran embryos (Fig.4A–C, black arrows), reinforcing our conclusion that the anterior-most neural structure was completely formed in the embryo. Interestingly, when the blastocoel roof was removed before ACE, at stages corresponding to type-A or -B, the dorsal structure was absent or formed only posteriorly, according to the timing of blastocoel roof removal (Fig.5). From these results, we concluded that the blastocoel roof at ACE contributes not to neural tissue but to ventral epidermis.

### The physical contact between prospective head neuroectderm and the leading edge tissue at ACE continues throughout the gastrulation process

**Figure 6:**
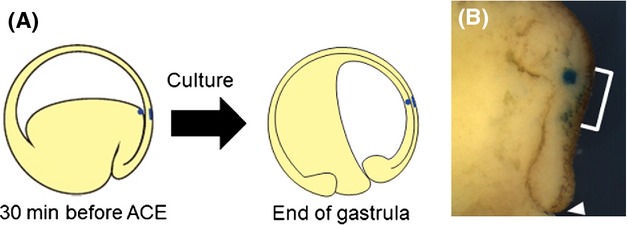
Prospective neuroectoderm and lining mesoderm are continuously associated with each other during gastrulation in *Cynops pyrrhogaster*. (A) Schematic illustration of the experimental procedure for internal and external labeling. At the beginning, the external surface of the equatorial region was labeled with Nile blue 30 min before anterior contact establishment (ACE) (6.5 h after blastopore appearance), and then a bead was placed in the internal tissue at the same location as the labeled external surface, which can be seen from the internal side. (A) If the internal and the external tissues do not slide in tandem, the bead is under the labeled tissue at the end of gastrulation. (B) A labeled embryo 24 h after blastopore appearance (sagittal section). White bracket indicates the Nile blue labeled region. The bead in the internal tissue was found below the surface layer labeled with Nile blue. White arrowhead in panel B indicates blastopore.

In *X. laevis*, it was shown that the anterior organizer tissue remained attached to the future head neuroectoderm during gastrulation (Koide *et al*. 2002). This was supported by the results of *C. pyrrhogaster* experiment (Fig.6). The outer surface of the dorsal-equatorial region of *C. pyrrhogaster* embryo at ACE, a region that corresponds to the prospective head neuroectoderm, was labeled with Nile blue. An agarose bead was placed on the leading edge tissue correctly lining the labeled ectoderm (Fig.6A), and the embryo was allowed to develop to observe the relative positions of the labeled tissue and the bead. At the end of gastrulation, the Nile blue labeled tissue and the agarose bead were in contact with each other (Fig.6B), strongly indicating that the leading edge tissue of *C. pyrrhogaster* is continuously associated with the prospective head neuroectoderm from ACE throughout gastrulation, as was observed in *X. laevis* gastrulation.

### Brachet's cleft is closely involved in the physical contact

In the previous paper, we showed that the *X. laevis* embryo at ACE had a just-formed Brachet's cleft (Koide *et al*. 2002). Observation of the internal morphology of other species’ embryos at ACE revealed that all the embryos had Brachet's cleft (Fig.2A–K). It is also shown that type A and B embryos in Figure5 had very poor Brachet's cleft, if any, when the blastocoel roof was removed (data not shown), but the type C embryo definitely had the well-formed Brachet's cleft.

Those observations underscored the importance of the formation of Brachet's cleft for ACE. If Brachet's cleft formation indicates the completion of ACE, the blastocoel roof should be dispensable for the neural formation when Brachet's cleft is just formed. In Eastern-Japanese common toad *Bufo japonicus formosus* and Miyako toad *Bufo gargarizans miyakonis*, the embryos are too dark to enable visualization of vital dye, and so the labeling experiments shown in Figure1 could not be performed. Brachet's cleft was observed 4 h after the time of blastopore appearance in both embryos (Fig.2G,H). To check whether anterior contact was established in the embryos 4 h after blastopore appearance, we removed the blastocoel roof from the embryos and allowed the embryos to develop to the tailbud stage (Fig.4F,G). We found that the resultant embryos had complete head structures with reduced ventral epidermises as observed in other amphibian embryos (5/5 in *B. japonicus formosus* and 2/2 in *B. gargarizans miyakonis*). The results suggest that Brachet's cleft formation may have a significance in relation to the physical contact in amphibian species.

### Anterior organizer is located at the dorsal blastocoel floor of the embryo at the onset of gastrulation

**Figure 7:**
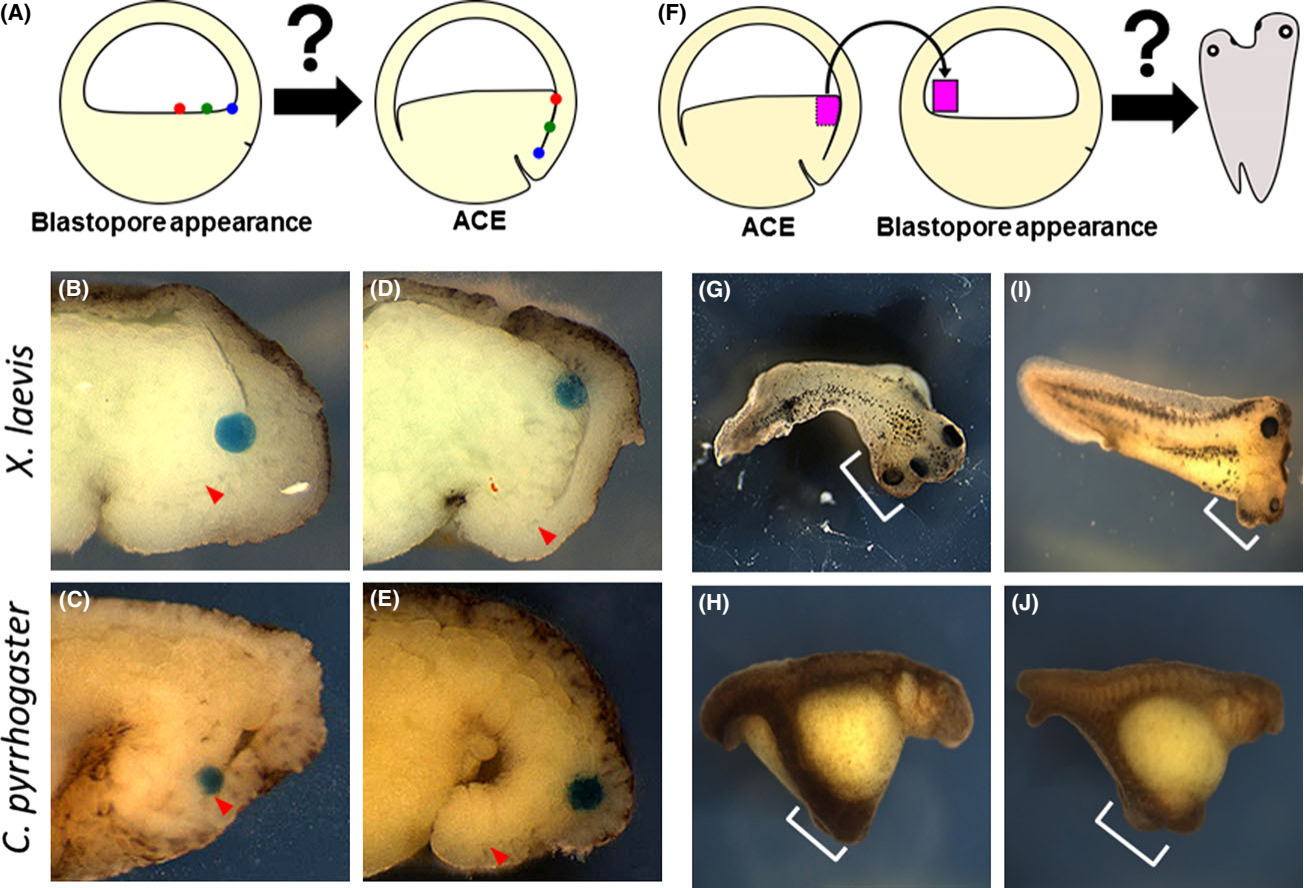
Not dorsal lip but dorsal blastocoel floor acts as anterior organizer in normal development. (A) Schematic representation of blastocoel floor tracing experiments. (B, C) A bead was placed the inside corner of blastocoel at blastopore appearance, and the embryo was dissected sagittally at ACE in *Xenopus laevis* and *Cynops pyrrhogaster*, respectively. (D, E) A bead was placed with a small distance from the corner in *X. laevis* and *C. pyrrhogaster*, respectively. Arrowheads indicate the tip of Brachet's cleft. (F) Schematic representation of the grafting assay. The embryo at blastopore appearance is grafted ventrally with blastocoel floor tissue of anterior contact establishment (ACE) embryo, and allowed to develop to the tailbud stage. (G, H) *X. laevis* and *C. pyrrhogaster* tailbud embryos, respectively. The embryos form a secondary axis containing head structures, as indicated by white brackets. (I, J) The embryo is grafted ventrally with the dorsal lip (Spemann organizer) tissue of ACE embryo, and allowed to develop to the tailbud stage.

It may be important to know how the Brachet's cleft is formed during gastrulation processes. Since it was suggested that Brachet's cleft is formed not by delamination but by the physical contact of blastocoel roof with blastocoel floor (Winklbauer & Schürfeld 1999), the tissue in contact with the prospective head neuroectoderm at ACE should be located at the dorsal blastocoel floor prior to ACE. To confirm this possibility, we placed an agarose bead on the blastocoel floor (Fig.7A). When we placed the bead close to the dorsal end of the blastocoel floor at the time of blastopore appearance, it ended up around the tip of Brachet's cleft at ACE (Fig.7B,C). The bead was also found in the middle of Brachet's cleft when it was placed on the blastocoel floor with a small distance from the dorsal end (Fig.7D,E) in both *X. laevis* and *C. pyrrhogaster*. The results confirm that the contact surface of Brachet's cleft is constructed between the blastocoel floor and the blastocoel roof in both amphibian species at the time of blastopore appearance. Taken together, the leading edge tissue is localized at the dorsal blastocoel floor at the time of blastopore appearance, and moves to establish physical contact with the prospective head neuroectoderm at ACE through the movement for Brachet's cleft formation.

If this inner tissue at the dorsal blastocoel floor is the anterior organizer, it should have the ability to induce a secondary axis when grafted ventrally. As expected, when we grafted the inner tissue (Fig.7F), 56% of *X. laevis* embryos (*n* = 16) and 27% of *C. pyrrhogaster* embryos (*n* = 15) had the secondary axes (Fig.7G,H). When the dorsal blastopore lip was transplanted, 51% of *X. laevis* embryos (*n* = 37) and 22% of *C. pyrrhogaster* embryos (*n* = 9) had the secondary axes (Fig.7I,J). Together, the results indicate that the dorsal blastocoel floor can act as a functional organizer in normal development.

### The physical contact is established by “subduction and zippering” (S&Z) movement

As the organizer equivalent tissue can be identified from the expression of its marker genes, we checked the expression pattern of *chordin* in *C. pyrrhogaster* and *X. laevis*. Careful observation of the shift of *chordin* expression pattern may help us to understand how the anterior organizer moves to establish the anterior contact.

**Figure 8:**
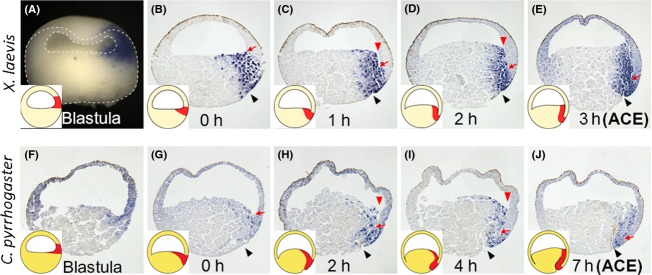
Expression patterns of *chordin* during early gastrulation in *Xenopus laevis* and *Cynops pyrrhogaster*. (A–E) Expression patterns of *X. laevis chordin* from late blastula to anterior contact establishment (ACE) (3 h after blastopore appearance). (F–J) Expression patterns of *C. pyrrhogaster chordin* from late blastula to ACE (7 h after blastopore appearance). (A, F) Expression in late blastula embryos. *Chordin* expression is first detected in the dorsal blastocoel roof. (B, G) Expression at the onset of gastrulation. *Chordin* is expressed not only in the surface layer (dorsal lip) but also internally in the blastocoel floor. (C, H) Expression in *X. laevis* 1 h after blastopore appearance, and in *C. pyrrhogaster* 2 h after blastopore appearance. A trench (red arrowheads) is formed between *chordin*-positive blastocoel floor and *chordin*-negative blastocoel roof. (D, I) Expression in *X. laevis* 2 h after blastopore appearance, and in *C. pyrrhogaster* 4 h after blastopore appearance. (E, J) Expression in ACE embryos (in *X. laevis* 3 h after blastopore appearance, and in *C. pyrrhogaster* 7 h after blastopore appearance). Insets are schematic drawings of the expression pattern. The developmental stages are shown at the bottom of each panel. Black arrowheads indicate blastopore. The outline of the embryo is shown by broken lines in panel (A). Note that the boundary between *chordin*-positive and -negative tissues corresponds to the bottom of the trench, and contributes to the tip of Brachet's cleft (red arrows).

*Chordin* is initially expressed in the dorsal blastocoel roof at the blastula stage in both species (Fig.8A,F; Kuroda *et al*. 2004) The *chordin*-negative ectoderm animally located relative to the organizer (*chordin*-positive) tissue should correspond to the prospective neuroectoderm, meaning that the prospective neuroectoderm and the organizer are aligned in tandem in the blastocoel roof of late blastula (Fig.9A), as indicated by the fate map of amphibian late blastula/early gastrula embryos (Vogt 1929; Nakamura 1942; Keller 1975).

The *chordin*-positive tissue moves vegetally and expands medially on the blastocoel floor (Fig.8B,G) by the time of blastopore appearance. At this stage, the *chordin*-positive tissue no longer exists in the blastocoel roof, and it turns out that the prospective neuroectoderm becomes oriented at an angle of approximately 90 degrees from the anterior organizer located in the blastocoel floor (Fig.9B). It may be important to remark that the boundary between the *chordin*-positive and -negative regions forms a pivot for the following movements. As the downward movement of the prospective neuroectoderm (*chordin*-negative ectoderm) progresses, the *chordin*-positive blastocoel floor is forcibly dragged down, forming a trench (Fig.8C,H). This trench-making movement resembles “subduction” (Fig.9B–D), which means one of the movements of plate tectonics. It may be noteworthy to say that the bottom of the trench corresponds to the boundary between *chordin*-positive and -negative tissues, and will contribute to the vegetal tip of future Brachet's cleft. Then, the *chordin*-positive side of the trench approaches the other side to make physical contact (Fig.8D,E,I,J). As a result, Brachet's cleft is formed from the posterior (vegetal) toward the anterior (equatorial), just like “zippering” (Fig.9D–F). The S&Z movement model shows the significance of Brachet's cleft for the proper function of the organizer.

## Discussion

In this study, we showed that the *X. laevis* gastrulation movement proposed previously (Koide *et al*. 2002) is applicable to a wide variety of amphibian species. Here we propose a unified model of amphibian gastrulation (Fig.10B).

In this model, the prospective organizer tissue is initially located in the blastocoel roof at the late blastula stage (Fig.8A, see also Kuroda *et al*. 2004). Since the prospective neuroectoderm is shown to locate in the animal hemisphere at the onset of gastrulation in the fate maps of various amphibian species (Vogt 1929; Nakamura 1942; Keller 1975), it follows that the prospective neuroectoderm and the organizer are aligned in tandem at the animal hemisphere prior to gastrulation (Fig.10B-1). Subsequently, the prospective neuroectoderm moves downward to expand the area of the organizer medially on the blastocoel floor by the time of blastopore appearance. This downward movement is also partially supported by the observation of MRI (Papan *et al*. 2007). Then, the prospective neuroectoderm and the anterior organizer area make a 90-degree angle at the blastocoel corner between the dorsal blastocoel floor and the blastocoel roof (Fig.10B-2). The “subduction” movement creates a trench between the two tissues (Fig.10B-2,3). Then, the “zippering” movement induces the physical contact via Brachet's cleft at ACE (Fig.10B-4,5). This S&Z movement could be seen in the live imaging of a single embryo by microscopic MRI (Papan *et al*. 2007). As this model clearly indicates that the prospective head region is specified at the early gastrula stage at the equatorial region, it follows that the axial structure should be progressively formed toward the posterior during the rest of the gastrulation movement. This prediction is supported by the results of time-lapse movies of the gastrulation of embryos embedded in gelatin to prevent the embryos from freely rotating due to the shift of the center of gravity (Movies S1 and S2). In the movies, as we expected, the blastopore lip starts closing from the position where the dorsal lip existed originally toward the ventral lip, so that the neural plate is formed on the vegetal surface (Fig.10B-6,7).

Kuroda *et al*. (2004) reported that the BCNE (Blastula Chordin- and Noggin-Expressing) center, which is defined by the region that expresses *chordin* and *noggin* at the blastula stage, contains both the prospective neuroectoderm and the Spemann organizer. In the report they claimed that the prospective neuroectoderm in the BCNE center expresses *chordin* when it was located at the blastocoel roof, and the expression disappears when it reaches the equatorial region. However, we found that the prospective neuroectoderm does not express *chordin* when it is located in the blastocoel roof at the time of blastopore appearance (Figs1, 5, 7 and 8). And the prospective neuroectoderm is found at the outer layer of the dorsal marginal zone at ACE. These results make us think that the BCNE center contains only the future organizer tissue and that the prospective neuroectoderm has never expressed *chordin*, and move downward from blastocoel roof to the equator to meet the anterior organizer at ACE.

The blastocoel floor derived tissue makes physical contact with the prospective neuroectoderm at the equator at the early gastrula stage (ACE), and the contact continues throughout gastrulation at the equatorial region (Fig.6, Koide *et al*. 2002). The earliest neural marker gene, such as Xotx2, is expressed at an earlier stage than the mid-gastrula stage (Blitz & Cho 1995). This blastocoel floor derived tissue could be regarded as a direct inducer of the anterior neuroectoderm, although it is well known that various signals emanating from a variety of tissues are necessary for the maintenance and patterning of the anterior neural tissues during development.

It has long been believed that the dorsal blastopore lip plays an important role in the organizer function (Spemann & Mangold 1924). The tissue located originally at the dorsal lip at the time of blastopore appearance was found in a region slightly farther from the prospective head neuroectoderm at ACE in both *X. laevis* and *C. pyrrhogaster* (Fig. S2B,C). This means that the dorsal lip derived tissue may never obtain an opportunity to directly contact the prospective neural tissue, and indicates that the dorsal blastopore lip may not function to induce anterior neural tissue in normal development, although the dorsal lip derived tissue actually has the ability to do so. As both the dorsal lip and the tissue at the dorsal blastocoel floor show the same expression patterns of various genes for organizer function, such as *chordin, goosecoid,* and *noggin* (Cho *et al*. 1991; Smith & Harland 1992; Sasai *et al*. 1994), it is not unusual that the dorsal lip is able to mimic the organizer function, even though it is not the functional organizer in normal development. The prospective head neuroectoderm and the anterior organizer are tissues derived from the blastocoel roof and the blastocoel floor, respectively, meaning that the contact surface has never been exposed to the external surface of the embryo at any time. Vogt's drawing supports this prediction (Fig. S3). Vogt labeled the surface of late blastula embryos with vital dye (Fig. S3A), grew the embryos up to the tailbud stage to see where the labeled tissues were localized, and found that the anterior portion of chordamesoderm remained unlabeled (indicated by the green bracket in Fig. S3B), although most of the stained tissue contributed to the internal axial tissue, including the pharyngeal endoderm and the posterior notochord (Vogt 1929). If our explanation is correct, the unlabeled tissue shown in Vogt's drawing should be labeled when the inner surface of the blastocoel cavity at the time of blastopore appearance is labeled (the green bracket in Fig. S3B). The result was what we expected (data not shown). This indicates that the organizer tissue can be divided into two regions: one is originally located at the blastocoel floor in early gastrulae and ends up as the anterior axial mesoderm including the prechordal plate, and the other is located at the surface of early gastrula and ends up as the posterior axial mesoderm. This may correspond to the upper blastopore lip because it is known that the dorsal lip involutes into the embryo at the early gastrula stage and contributes to the axial mesoderm (Shih & Keller 1992; Winklbauer & Schürfeld 1999), though the bottle cells are known to come to lie in the anterior archenteron (Kaneda & Motoki 2012)

In this report, we show that the anterior-most axial mesoderm and the anterior neuroectoderm are in contact with each other from the time of ACE through the end of gastrulation (Fig.10B-6). But the anterior migration of the leading edge after ACE is definitely observed, as many researchers have reported (Nakatsuji *et al*. 1982; Boucaut *et al*. 1984; Winklbauer & Nagel 1991; Johnson *et al*. 1993). In our model, we propose that the leading edge tissue consists of the predicted organizer (*chordin*-expressing cells) from the time of blastopore appearance through ACE, and after ACE, the prospective endodermal tissue precedes the anterior terminus of the axial mesoderm. This means that different cells contribute to the leading edge before and after ACE (Fig.10B-6). It was also reported that the dorsal blastocoel floor of early gastrula embryo ends up as the pharyngeal endoderm (Winklbauer & Schürfeld 1999). This may suggest that only the dorsal-most portion of the blastocoel floor prior to ACE functions as the organizer, and another part of the blastocoel floor ends up as the pharyngeal endoderm, or the dorsal-most portion of blastocoel floor after ACE contributes to the pharyngeal endoderm. Indeed, it is known that a tissue of the anterior archenteron roof including the leading edge tissue of the late gastrula does not express marker genes of the axial mesoderm (such as *chordin* and *goosecoid,* Yamaguti *et al*. 2005; Kaneda & Motoki 2012).

We showed that the leading edge tissue can induce the secondary axis as well as the dorsal lip (Fig.7F–J). However, Bouwmeester *et al*. (1996) showed that the leading edge tissue is a rather weak inducer of the secondary axis. This contradiction might be caused by the difference in embryonic stages. Since it takes very short time from the time of blastopore appearance to ACE, it is difficult to know exactly the embryonic stage from the external morphology. Moreover, it is said that the external morphology does not necessarily reflect the internal morphology. Therefore, the leading edge tissue used in their experiments may be slightly different from what we used. The important thing is that “the anterior contact” of the anterior axial mesoderm with the head neuroectoderm is preserved until the end of gastrulation, and they never slide from each other. Therefore the leading edge tissue may be the mesoderm or the endoderm depending on the developmental stage.

Although limited anterior migration of the head mesoderm was reported recently for a few amphibian species (Poznanski & Keller 1997; Kaneda & Motoki 2012), it is still widely believed that the axial mesoderm migrates animally during amphibian gastrulation. This confusion might make it difficult to construct a general model of amphibian gastrulation. We propose herein a unified model of amphibian gastrulation, which may allow us to directly compare the gastrulation movements of the other chordates. In the previous paper (Koide *et al*. 2002), we indicated that a direct comparison of gastrulation movements between amphibians and amniotes might be possible at this point. Here we would like to propose a similarity between amphibian gastrulation and that of a basal chordate, amphioxus (Fig. S4). For example, basal chordates, such as cephalochordates (amphioxus) or tunicates (ascidian), form simple tadpole larvae with a small number of cells, and share the same basic body plan with vertebrates (Jeffery 1992; Yu *et al*. 2007). Those embryos undergo simple invagination during gastrulation with little involution, and the presumptive mesoderm cells fold into the depression (Zhang *et al*. 1997; Munro *et al*. 2006). This gastrulation manner closely resembles the S&Z movement in amphibian. At the blastula stage, the organizer (presumptive notochord) and the prospective neuroectoderm are aligned in tandem in the surface layer (Fig. S4B-1). At the onset of gastrulation, one side of the blastula is flattened, and the organizer moves medially (Fig. S4B-2,3). Subsequently, the embryo undergoes simple invagination with little involution, and then the presumptive notochord cells fold into the depression (Fig. S4B-4,5). At last, the organizer reaches the anterior tip of the prospective neuroectoderm. We propose that this protochordate gastrulation shows the S&Z movement. Compared with the model of amphibian gastrulation, we may be able to construct a common model of chordata gastrulation.

## Supporting Information

**Fig. S1.**Rate of each type in the experiment of neutral red injection into blastocoel roof. **Fig. S2.** Not dorsal lip but dorsal blastocoel floor acts as anterior organizer in normal development. **Fig. S3.** Anterior axial tissue is prevented from labeling surface cells at blastopore appearance. **Fig. S4.** Proposed model of amphibian gastrulation is comparable to protochordate gastrulation.

**Movie S1.**Time-lapse movie of vegetal view of *Rana nigromaculata* embryo. **Movie S2.**Time-lapse movie of vegetal view of *Xenopus laevis* embryo.
