# Supplementary material for: The Spemann organizer meets the anterior-most neuroectoderm at the equator of early gastrulae in amphibian species
Source: Dev Growth Differ. 2015 Mar 10;57(3):218–31. doi: 10.1111/dgd.12200 (PMC4402005; doi:10.1111/dgd.12200)
Supplement: Supplementary file 1 — Fig. S1.Rate of each type in the experiment of neutral red injection into blastocoel roof. Fig. S2. Not dorsal lip but dorsal blastocoel floor acts as anterior organizer in normal development. Fig. S3. Anterior axial tissue is prevented from labeling surface cells at blastopore appearance. Fig. S4. Proposed model of amphibian gastrulation is comparable to protochordate gastrulation. [file dgd0057-0218-sd1.zip › Legends of Supplementary Figs (#12200).docx]

**Supplementary Fig. 1.** **Rate of each type in the experiment of neutral red injection into blastocoel roof.**

Line graphs of neutral red injected embryos of type A (blue), type B (red), and type C (green) in *B. orientalis* **(A)**, *R. japonica* **(B)**, *R. porosa brevipoda* **(C)**, *R. rugosa* **(D)**, *S. tropicalis* **(E)**, *X. laevis* **(F)**, *A. mexicanum* **(G)**, *C. ensicauda* **(H)**, *C. pyrrhogaster* **(I)** and *H. nebulosus* **(J)**. Vertical axis indicates rate of each type (%). Horizontal axis indicates injection time. In graphs **A-D and F-I**, the horizontal axis means “hours after blastopore appearance.” In graph **E**, the horizontal axis means “minutes after blastopore appearance.”

**Supplementary Fig. 2.** **Not dorsal lip but dorsal blastocoel floor acts as anterior organizer in normal development.**

**(A)** Schematic representation of dorsal lip labeling experiment. **(B-C)** Lineage tracing of the dorsal lip. **(B)** The dorsal lip of *X. laevis* embryo was labeled with Nile blue at blastopore appearance, and the embryo was dissected sagittally at ACE. **(C)** The same experiment in *C. pyrrhogaster*. The labeled cells end up not at Brachet’s cleft but at the anterior tip of archenteron in both *X. laevis* and *C. pyrrhogaster*. Red arrowheads indicate the tips of Brachet’s cleft, and white brackets indicate the prospective head neuroectoderm.

**Supplementary Fig. 3. Anterior axial tissue is prevented from labeling surface cells at blastopore appearance.**

Fate mapping experiment performed by Vogt. Arrowhead indicates the prospective blastopore region. The stage is **(A)** blastula and **(B)** tailbud, respectively. Green bracket in **(B)** indicates the portion of anterior axial tissue that could not be stained.

**Supplementary Fig. 4. Proposed model of amphibian gastrulation is comparable to protochordate gastrulation.**

**(A)** Proposed model of amphibian gastrulation. The organizer and the prospective neuroectoderm are represented by red and blue, respectively. **(B)** Model of protochordate gastrulation. The organizer (presumptive notochord) and the prospective neuroectoderm are represented by red and blue, respectively.
