# Supplementary material for: The Spemann organizer meets the anterior-most neuroectoderm at the equator of early gastrulae in amphibian species
Source: Dev Growth Differ. 2015 Mar 10;57(3):218–31. doi: 10.1111/dgd.12200 (PMC4402005; doi:10.1111/dgd.12200)
Supplement: Supplementary file 1 — Fig. S1.Rate of each type in the experiment of neutral red injection into blastocoel roof. Fig. S2. Not dorsal lip but dorsal blastocoel floor acts as anterior organizer in normal development. Fig. S3. Anterior axial tissue is prevented from labeling surface cells at blastopore appearance. Fig. S4. Proposed model of amphibian gastrulation is comparable to protochordate gastrulation. [file dgd0057-0218-sd1.zip › Supplemental Figure4 (#12200).pptx]

## Slide 1
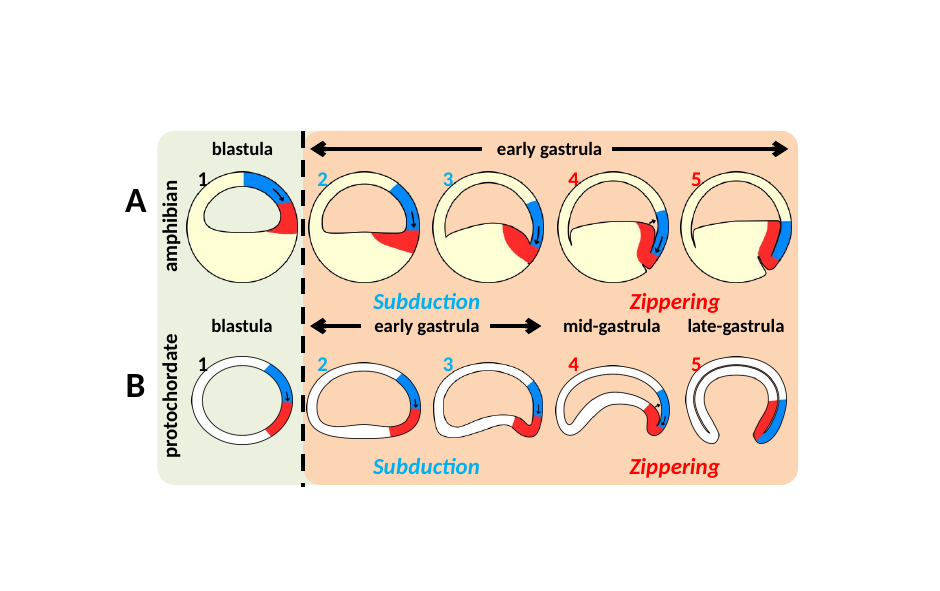

blastula
early gastrula
1
2
3
4
5
A
amphibian
Subduction
Zippering
early gastrula
mid-gastrula
late-gastrula
blastula
2
1
3
4
5
B
protochordate
Zippering
Subduction
